# Lipid-Based Nanomaterials for Drug Delivery Systems in Breast Cancer Therapy

**DOI:** 10.3390/nano12172948

**Published:** 2022-08-26

**Authors:** Lekshmi Rethi, Chinmaya Mutalik, Dito Anurogo, Long-Sheng Lu, Hsiu-Yi Chu, Sibidou Yougbaré, Tsung-Rong Kuo, Tsai-Mu Cheng, Fu-Lun Chen

**Affiliations:** 1 International Ph.D. Program in Biomedical Engineering, College of Biomedical Engineering, Taipei Medical University, Taipei 11031, Taiwan; 2 International Ph.D. Program for Cell Therapy and Regeneration Medicine, College of Medicine, Taipei Medical University, Taipei 11031, Taiwan or; 3 Faculty of Medicine and Health Sciences, Universitas Muhammadiyah Makassar, Makassar City 90221, South Sulawesi, Indonesia; 4 Graduate Institute of Biomedical Materials and Tissue Engineering, College of Biomedical Engineering, Taipei Medical University, Taipei 11031, Taiwan; 5 Department of Radiation Oncology, Taipei Medical University Hospital, Taipei Medical University, Taipei 11031, Taiwan; 6 Ph.D. Program for Translational Medicine, College of Medical Science and Technology, Taipei Medical University, Taipei 11031, Taiwan; 7 Institut de Recherche en Sciences de la Santé/Direction Régionale du Centre Ouest (IRSS/DRCO), Nanoro BP 218, 11, Burkina Faso; 8 Graduate Institute of Nanomedicine and Medical Engineering, College of Biomedical Engineering, Taipei Medical University, Taipei 11031, Taiwan; 9 Graduate Institute of Translational Medicine, College of Medicine and Technology, Taipei Medical University, Taipei 11031, Taiwan; 10 Taipei Heart Institute, Taipei Medical University, Taipei 11031, Taiwan; 11 Department of Internal Medicine, Division of Infectious Diseases, Taipei Municipal Wan Fang Hospital, Taipei Medical University, Taipei 11031, Taiwan; 12 Department of Internal Medicine, School of Medicine, College of Medicine, Taipei Medical University, Taipei 11031, Taiwan

**Keywords:** drug delivery system, conjugation, targeting, breast cancer, liposomes, exosomes, micelles, safety, efficacy

## Abstract

Globally, breast cancer is one of the most prevalent diseases, inducing critical intimidation to human health. Lipid-based nanomaterials have been successfully demonstrated as drug carriers for breast cancer treatment. To date, the development of a better drug delivery system based on lipid nanomaterials is still urgent to make the treatment and diagnosis easily accessible to breast cancer patients. In a drug delivery system, lipid nanomaterials have revealed distinctive features, including high biocompatibility and efficient drug delivery. Specifically, a targeted drug delivery system based on lipid nanomaterials has inherited the advantage of optimum dosage and low side effects. In this review, insights on currently used potential lipid-based nanomaterials are collected and introduced. The review sheds light on conjugation, targeting, diagnosis, treatment, and clinical significance of lipid-based nanomaterials to treat breast cancer. Furthermore, a brighter side of lipid-based nanomaterials as future potential drug delivery systems for breast cancer therapy is discussed.

## 1. Introduction

Nanomaterials have been extensively applied in various fields, such as medicine, materials science, and energy application [1,2,3,4,5,6,7,8,9]. Recently, cancer nanotechnology has been developed to treat cancer based on nanomaterials. As the years proceeded, the field of cancer nanotechnology has gained a great foothold because of its great prospects to improve the treatment of cancer [10]. Breast cancer is the second most commonly occurring cancer, with over 2 million new cases in 2018 [11,12,13,14,15]. Breast cancer starts from the cells of the lobules, extending to the nipples by the passage of milk [16]. The vascular supply and strong interstitial pressure narrow down the scope of drug penetration successfully. The slow diffusion of the drugs is the main cause and this is where the nanoformulations come in handy [17,18,19,20,21]. There are different nanoformulations used in these for targeted delivery in the field of cancer nanotechnology. One of the most focused of this array of formulations is lipid formulations, which are easy to produce, biocompatible, and open to changes [22]. This drug delivery system has several positive aspects, such as increased drug concentration of tumor cells, which leads to damage in the normal tissues around the tumor. Various organic and inorganic nanomaterials have been developed as nanocarriers for the treatment of breast cancer [23]. The low toxicity, lack of immune system activation, the capability to carry hydrophilic and hydrophobic drugs, biocompatibility, and biodegradability make liposomes the active agents for the carrier system [24,25,26].

Liposomes have passed clinical trials as they showed tremendous potential as a carrier in therapeutics. The advantages of lipid-based drug delivery systems are that they improve both the pharmacokinetic and pharmacodynamic profile of the drug, showing controlled release of the drug with less toxicity [27,28]. Surface modification has increased systematic circulation, accumulation in target sites, and specified drug delivery of the liposomes [29,30]. Exosomes are used as targeting agents and are now used for treating cancers. With ideal sizes of 30–120 nm for the delivery, exosomes are secreted from the cells. They are biocompatible, permeable, and have low toxicity and immunogenicity, even when they are encapsulated with siRNAs and drugs. These are used to treat cancers that are hard to treat [31,32].

## 2. Conjugation and Targeting Moieties of Liposomes

### 2.1. Liposomal Nanomaterials

Lipid-based nanomaterials of liposomes are bilayer spherical vesicles of phospholipids, the first lipid-based nano-drug carrier system. It was first published in 1965 by Bangham et al. that univalent cations and anions were able to diffuse out by spontaneously similarly forming liquid crystals of lecithin as the diffusion of ions across a biological membrane [33]. This lecithin was later known as a liposome. These made their own space in drug delivery and cancer treatment as they display zero toxicity, capable of transporting hydrophobic as well as hydrophilic molecules, and they are good at increasing the prolonged half-life and controlled release of drugs, which improves the action of the drug. To improve the solubility and to avoid the invasion of immune cells, the liposomes are modified usually by PEG (polyethylene glycol). When the size is considered, it ranges from 0.5 to 10 nm (multilaminar vesicles), 10 to 100 nm (small unilamellar vesicles), and larger than 100 nm (large unilamellar vesicles).

There are several cytotoxic drugs, such as carboplatin, cisplatin, cytarabine, dacarbazine, irinotecan, oxaliplatin, paclitaxel, docetaxel, doxorubicin (DOX), ecteinascidin, etoposide, fluorouracil, gemcitabine, and pemetrexed, used in the nanoformulation. Among all these drugs, DOX was the first drug to be conjugated with the liposome for clinical practice. In the treatment of cancer, liposomes are exceptionally useful as they have the ability to reduce the toxic side effects of chemotherapeutic drugs. The passive and active targeting of cancer can be conducted by different strategies using different drugs in the liposomes, which improve the specificity only to cancer cells. By the liposomal encapsulation, the drug clearance by the immune and renal system and the circulation time of the drugs can be increased with maximum effect on the tumor cells. As mentioned earlier, liposomes can transport the hydrophobic and hydrophilic drugs by increased stability and solubility.

Liposomes can enter the tumor cell through passive targeting, while active targeting has a structurally modified liposome with antibodies, which can exclusively target only tumor cells. There is another method in which liposomes are made with stimulus-sensitive structures, where temperature, pH, and magnetic fields are parameters for controlled delivery of the drugs on the external trigger [34]. Nanomaterials loaded with DOX are used for the treatment of breast cancers. The nanoparticle, which is loaded with DOX, was found to show a high rate of cellular uptake and accumulation on the tumor tissue with reduced cardiac and gastrointestinal toxicity. The liposome loaded with DOX has reached clinical trials now. The DOX-loaded liposome combined with lapatinib is used for HER2-positive breast cancer patients, reaching phase 1b. The treatment of metastatic breast cancer with cyclophosphamide (CM) or vinorelbine (MV), co-administered with Myocet, has reached phase III trials [35]. Extensive studies have been implemented for targeting tumors by using liposomes [28,29,30]. The recent advances in tumor targeting by using liposomes is by recognizing biomarkers and the release of the drug by the stimulus.

### 2.2. Liposomes as Drug Carriers

Tumor targeting using drug-loaded liposomes depends on the pathophysiological property of the tumor, called passive targeting. The plus point of liposomes is that they can retain low-molecular-weight drugs at the tumor site for a longer time than other nanomaterials [36,37,38]. For protecting the liposomes from adverse conditions, there are different measures taken. The PEG is coated using steric stabilization over the surface of the liposome. This will produce a hydrophilic layer over the surface, thus, preventing aggregation and blood component interactions. When the liposomes are conjugated with the PEGs, they protect them from the reticuloendothelial system, as well as the longevity in the blood, as the systematic circulation in blood blocks liposomes from reaching the target sites. The PEG shields the surface charge, by which it prevents opsonization, which, in turn, enhances the interaction of the blood and liposomes. The PEG-coated liposomes reduce uptake of the macrophages and increase circulation time [39,40]. The EPR effect is another parameter of passive targeting. The particular cancers have a different endothelial gap, making the size an important factor. The liposomes should have a size smaller than 400 nm and more effective extravasation occurs when the size is less than 100 nm [41,42].

### 2.3. Active Targeting Liposomes

The liposomes are often designed in a way to target specific sites. These are made by conjugating moieties, including ligands, peptides, and Mab, onto the surface of the ligands [43,44]. An example of this is the folate and transferrin receptors, which are overexpressed in cancers and are made use of for modifying the liposomes to be tumor specific. These liposomes, which are accumulated in the tumor microenvironment, are often endocytosed into the cells by the surface receptors. For the efficient targeting of cancer cells, the targeting moiety is linked in a sufficient range, which has an ideal affinity towards the cell receptors on the surface. Therefore, a wide range of tumor-specific targeting ligands is used [44,45,46].

### 2.4. Cell Surface Receptor Targeting

For a great therapeutic response, the targeting liposomes should bind to specific cell surface receptors. Increasing the drug delivery to the targeting cells is often achieved by the attachment of the liposomes to the surface receptors. These are often achieved by the ligands or antibodies, which are specific to these cell receptors. As mentioned earlier, the folate and transferrin receptors are highly specific to certain receptors to the cancer cells [46,47].

### 2.5. Targeting Transferrin Receptor (TfR)

The expression of TfR is higher in tumor cells and has an association with iron for cancer cell proliferation. This helps the targeting of these receptors using TfR-targeted liposomes for anticancer therapy. The doxorubicin-encapsulated TfR-targeted liposomes showed improved intracellular uptake, biodistribution of the drug, and pharmacokinetic profile compared to normal liposomes [48,49,50]. The studies showed that TfR-targeted liposomes can be used as a targeted therapy for the NSCLC, head-neck cancer, breast cancer, etc. [51].

### 2.6. Targeting Epidermal Growth Factor Receptor (EGFR)

EGFR is the most commonly targeted receptor in many cancers as it helps in proliferation, angiogenesis, and metastasis. The liposome is a conjugated EGFR-targeted Mab, which has high specificity and specific drug delivery [28]. HER-2, which is encoded with EGFR, is overexpressed in breast cancer for about 20%. Not only are they seen overexpressed in breast cancer but also in brain, lung, bladder cancers, etc. The HER-2-targeted PEGylated liposomal doxorubicin has been reported to have a greater accumulation in tumor cells [52]. Trastuzumab conjugated to a maleimide-PEGylated liposome down-regulated the expression in the breast cancer cell line [53]. Even though they have all these good sides, they have several challenges to face. They are the ligand/target affinity, the receptor quantity in the cell surface, and the PEG acting as a barrier for the receptor–ligand interaction. The high drug:ligand ratio in the immune liposome helps in the delivery of the drug with a few ligands. This helps in the increased uptake and signaling properties of Ab fragments. The high-density ligands on the liposomes increased binding to target cells but the removal through circulations made them less localized in the tumor. This is usually overcome by the PEGylation process.

### 2.7. Targeting Folic Acid (FA) Receptor

FA is used as the targeting ligands for cancers, such as ovarian, kidney, breast, lung, colon, and brain, as the folic acid receptors are overexpressed in these cancers. Not only these receptors are used in cancer treatment but they are also used for treating inflammatory diseases. Receptor-mediated endocytosis is the mechanism where folic-acid-conjugated liposomes are used for drug delivery. The folate-conjugated liposomes are made by modifying the surface of the liposome by the PEG. This will help with the entrance of the liposomes in the cancer cells. Studies are being conducted, in which doxorubicin-conjugated liposomes for FA-receptor-expressing cancer showed higher uptake (45-fold) than non-targeted liposome and the cytotoxicity is higher than the unmodified plain liposome [54,55,56,57,58]. The folic acid receptor targeting liposomes is used for gene delivery as a form of lipoplex. It promotes cell death and reduces tumor growth [59]. The FA-coupled liposomal nanoparticle is also used for drug delivery, gene transduction, and as a chemotherapeutic agent. This varied usefulness makes it a great targeting moiety [60].

### 2.8. Carcinoembryonic Antigen-Like Cell Adhesion Molecules (CEACAMs)

CEACAM6 is an intercellular adhesion molecule that is overexpressed in a wide variety of human cancers, including breast cancer, pancreatic cancer, and lung cancer [61,62,63], and is associated with tumorigenesis, tumor cell adhesion, invasion, and metastasis. CEACAM6 expression associated with cancer cell proliferation, migration, invasion, and angiogenesis, plays an important role in several cancers’ progression, tumorigenesis, tumor cell adhesion, invasion, and metastasis. Currently, CEACAM6 is applied for breast-cancer-targeted treatment and diagnosis for breast cancer [64]. Liposomes are widely used as drug delivery systems or image payload vehicles [65].

### 2.9. Preparation of Antibody-Conjugated Liposome

Simple adsorption is a basic approach for conjugating the antibody with the liposome. This is often viewed as an intentional coupling method or undesirable side reaction. Weissmann and coworkers reported that aggregated immunoglobulins can coat and partially insert into liposomes with the Fc region exposed to the surrounding medium. During or after sonication, the antibodies can be absorbed into the SUV. If insertional or ionic mechanisms are involved, the adsorption is greater in small negatively charged liposomes. It is also to be noted that the presence or absence of the lipid during sonication of the antibody has no significant effect on the binding capacity. Senior et al. highlighted adsorption after they completed the large non-specific binding of native Ab to liposomes, which has a covalent bonding with thiolated Ab. There was no reported leakage of contents after the binding of 34–89% Ab over the surface. The main attraction of the Ab-coated liposome is extreme stability in the presence of serum. Two major approaches are developed for the specific and controlled coating of liposomes with the antibody. The primary approach is to reroute the lipid amino group (especially phosphatidylethanolamine), which will covalently bind the Ab with the liposome during conjugation. The secondary approach is to transform the hydrophilic Ab to an amphipathic one for non-covalent insertion into the liposomal bilayer [66].

### 2.10. Antibody Conjugation Method

#### 2.10.1. Amine Modification

The modification of amine groups is a common procedure for Ab-Liposome conjugation. The EDC is the procedure usually used for liposomes with carboxylic acid. As these are condensing agents, they form protein–protein polymers. These reactions are difficult to control, as a separation of the protein and liposome can occur [67,68,69]. Fatty acids, such as NHS, are used for modifying Ab before they are conjugated with liposomes. An EDC–NHS combination is also used for activating acid groups on liposomes to be bonded with the Ab amino group [70,71]. Crosslinkers, such as SPDP, S-acetylthioglycolic acid N-hydroxysuccinimide ester (SATA), and 4-(p-maleimidophenyl)butyric acid N-hydroxy– succinimide ester (SMPB), are used for direct reaction on the thiolated and maleimide Ab. These reagents should be removed as these can cause coupling reactions and also damage the disulfide bond [72,73,74,75].

#### 2.10.2. Carbohydrate Modification

When the amino group modification damages the disulfide bond of the Ab, the carbohydrate modification of the Ab with sodium periodate forms an aldehyde group, which, in turn, helps the protein–liposome conjugation [76]. According to the class of the Ab, the modification by glycosylation is achieved, usually in IgG type Ab, in the CH_2_ region of the heavy chain. These affect the function, such as Fc receptor binding and complement activation, but do not affect the binding to Ag [77]. Even though these are highly useful, the approach is not common. The studies are limited to the acyl hydrazides in liposomes conjugated to IgM, Lipid-PEG-Hydrazide used for conjugating IgG-Liposome conjugations, liposome–liposome crosslinking, etc. [77,78,79].

#### 2.10.3. Disulfide Bonds

As mentioned earlier, the cleavage of the disulfide bonds in Ab (especially Fab or Fab2) is performed by DTT or 2-mercaptoethylamine. The cleavage for the disulfide bonds can cause a lack of binding activity as these are responsible for the maintenance of the variable region structure [77].

## 3. Lipid-Based Drug Delivery Systems and Treatment

### 3.1. Liposomes

To date, there is a requirement for a better drug delivery system to make the treatment and diagnosis easily accessible to patients. Targeted drug delivery systems are of great significance in the present scenario for easy internalization of drugs to treat diseases efficiently and the safety of drug delivery system design and synthesis used are more significant [80,81,82]. Liposomes belong to the class of lipids known to overcome drug solubility, toxicity, and drug delivery system challenges in the future. In the present scenario, liposomes and related liposome-conjugated particles are lipid bilayer particles and are extensively studied for the safe transport of drugs to infected sites, targeted drug delivery, and breast cancer treatment [83]. In recent advances, hydroxyurea (HU), a chemotherapeutic agent in use, has toxic effects; to overcome the problem, HU was coated with nanoliposomes, called nanoliposome containing HU (NL-HU). NL-HU was found to be a biocompatible drug carrier, showed drug uptake enhancement, and tested against BT-474 breast cancer cell lines. This in vitro model showed potential for further in vivo and clinical advancement in breast cancer treatment [84]. Designing drug-carrying liposomes plays a vital role in drug delivery systems and therapies. To address designing issues with liposomes and overcome leakage of the drug through liposomes, recently, nanobowl-supported liposomal doxorubicin (DOX@NbLipo) was synthesized for an efficient, stable, and safe drug delivery system [85].

**Figure 1:**
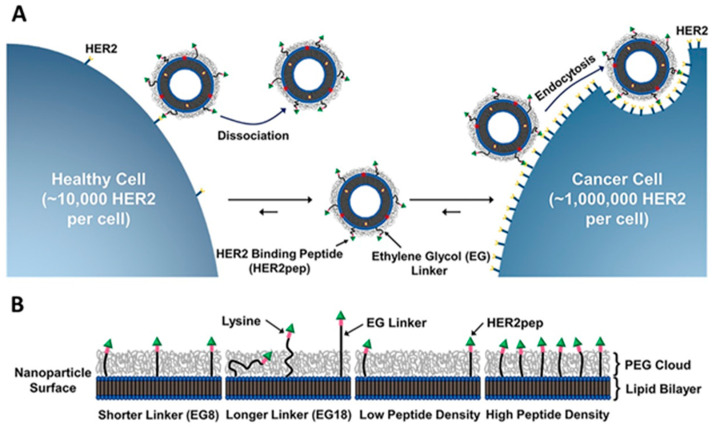
Schematic representation of specific targeted drug delivery strategy of Dox prodrug-loaded TNPHER2pep for HER2 overexpressing breast cancer cells. (**A**) Dissociation and association of expression of HER2 on epithelial cells within breast cancer lesions are many folds higher than that on healthy epithelial cells. On HER2 overexpressing breast cancer cells, TNPHER2pep liposome undergoes endocytosis (**B**) density of peptide and length of EG linker are designed, synthesized, and applied using both in vitro and in vivo methods to increase liposome cellular uptake in breast cancer cells. Reproduced with permission from ref. [86] Copyright © 2020, Elsevier.

Kim et al. reported peptide-targeted liposomal doxorubicin nanomaterials recently known to have inhibition potential against human epidermal growth receptor 2 (HER2)-positive breast cancer in vitro as well as in vivo [86]. The mechanism of action was proposed to target the HER2 protein, which increases in number on the cell wall of cancer cells, increasing the tumor-specific targeted delivery of the drug. In Figure 1, targeted nanomaterials (TNPs) were designed to have a specific density of peptide and the length of conjugator, manipulation of peptide density, and linker length indicated TNP-associated DOX delivery to HER2 receptors of breast cancer cells to be effective in specific drug delivery and treatment in vitro as well as in vivo. In this study, researchers used two kinds of linkers, ethylene glycol 18 and ethylene glycol 8 (EG-18 and EG-8). Moreover, the study is concentrated on the specific HER2 receptor protein targeted by peptide-associated TNPs, as the number of HER2 receptors increases the peptide-associated liposome containing DOX bind to specific HER2 receptor sites in the breast cancer cells, and the targeting peptide dissociation half-life is significantly lower; only the breast cancer cells are targeted by liposomes and undergo endocytosis in cancer cells and were found to eliminate the cancer cells by releasing DOX (Figure 1A,B). The liposomal design and peptide engineering used in this study were found to be effective in the elimination of breast cancer tumors with specificity in vitro and in vivo, opening the gate for further clinical evaluation in HER2-positive breast cancer [87].

**Figure 2:**
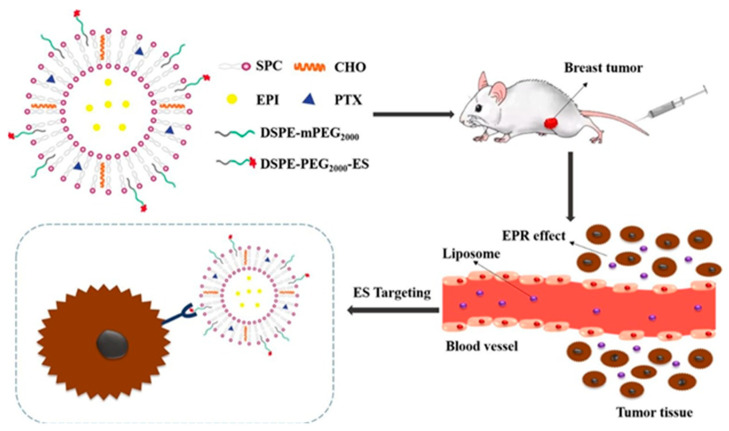
Schematic representation of the liposomal drug delivery carrier system and treatment. The overexpressed estrogen receptors on cancer cells were targeted by estrone in vitro and in vivo. Reproduced with permission from ref. [88] Copyright © 2020, Elsevier.

Another recent example is of a liposomal drug delivery system carrying a combination of two drugs, epirubicin (EPI) and paclitaxel (PTX), pegylated by estrone (ligand) to target the estrogen receptor in breast cancer treatment (Figure 2) [88]. In Figure 2, taking the advantage of overexpression of the estrogen receptor in breast cancer, the mechanism of action was proposed with regard to a receptor–ligand pathway in vitro as well as in vivo. Furthermore, as described earlier, there is an overexpression of estrogen receptors in the case of breast cancer and estrone (ES), which can reach the target-specific site as proposed in this study, and the combination of two drugs, EPI and PTX, contained in the liposome were found to be effective in the elimination of an MCF-7 breast cancer cell line and tumor in a mouse model. The mice in the experiment were intravenously injected with ES steric-stabilized liposome containing EPI and PTX drugs (ES-SSL-EPI/PTX) to treat the MCF-7 tumor and found the ES-SSL-EPI/PTX to be effective in the inhibition of tumor growth and biocompatible with the surrounding environment of the tumor. The liposomal drug delivery system, ES-SSL-EPI/PTX, was found to be effective in breast cancer treatment in vitro, in vivo, and opening the gate for further clinical evaluations.

**Figure 3:**
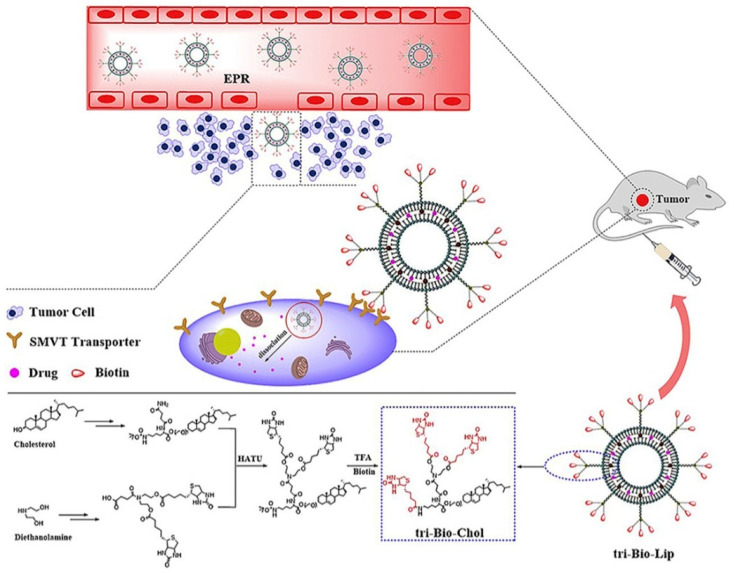
Schematic representation of the tri-Bio-Lip effective drug delivery system to inhibit two kinds of cancer cell lines, mice breast cancer cell line (4T1) and human breast cancer cell lines (MCF-7), respectively. Reproduced with permission from ref. [89] Copyright © 2020, Elsevier.

In recent advances, one of the studies reported the effectiveness of biotin and branched biotin-linked liposomes containing PTX drugs targeting specifically sodium-dependent multivitamin transporter (SMVT) receptor protein, which can be overexpressed in breast cancer [89]. Biotin-linked liposomes were known to target SMVT receptors, which are overexpressed in breast cancer. In the study, researchers were able to synthesize four types of branched biotin-linked liposomes (Bio-Lip, Bio-Bio-Lip, tri-Bio-Lip, and tetra-Bio-Lip). The drug delivery system used in this case was a liposome–ligand pathway. In Figure 3, the drug PTX was encapsulated with biotin-linked liposome and, further, the liposomes were branched. The tri-Bio-Lip was found to be more effective than its counterparts. The tri-Bio-Lip was found effective in the inhibition of two kinds of cancer cell lines, mice breast cancer cell line (4T1) and human breast cancer cell lines (MCF-7), respectively. The targeting ability and inhibition efficacy of tri-Bio-Lip towards tumor-bearing BALB/c mice was found to more effective than the counterparts Lip, Bio-Bio-Lip, and tetra-Bio-Lip. The tri-Bio-Lip was found to be more effective in targeting SMVTs and suppression of breast cancer in vitro and in vivo. This study further considers the clinical evaluation subjected to its biocompatibility.

**Figure 4:**
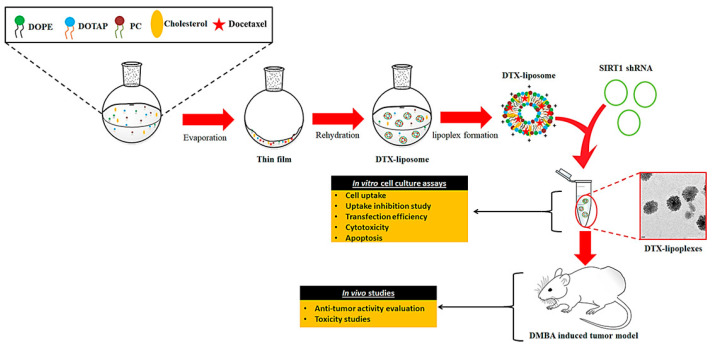
Schematic representation of DTX-Lipoplex pH-responsive nano-drug delivery system in vitro and in vivo. Reproduced with permission from ref. [90] Copyright © 2020, Elsevier.

Swami et al. recently reported pH-sensitive liposomes carrying docetaxel (DTX), SIRT1, and shRNA to be more effective in the treatment of breast cancer and biocompatible for normal cells [90]. In Figure 4, the researchers synthesized liposomes using 1, 2-dioleoylsn-glycero-3-phosphoethanolamine (DOPE), 1,2-Dioleoyloxy-3-trimethylammoniumpropanchloride (DOTAP), phosphatidylcholine (PC), and cholesterol. The synthesis was further incorporated by SIRT1 and shRNA to make pH-responsive liposome (lipoplex) and loaded with docetaxel (DTX-Lipoplex). A DTX-Lipoplex drug delivery system was designed to respond to changes in pH so that once it reaches the site of action, it can release DTX to suppress breast cancer cells and tumors in the microenvironment. The DTX-Lipoplex was found to be effective in inhibiting breast cancer in vitro (~3-fold more) as well as in vivo (~78% tumor burden) compared to the currently marketed Taxotere,^®^ which is a non-pH-responsive agent. The DTX-Lipoplex pH-responsive nano-drug delivery system was found to be a peculiar system, biocompatible, and opens the gate for further clinical evaluations. Recent examples of liposome-based drug delivery systems for breast cancer therapy have been collected in Table 1.

### 3.2. Exosomes

In the context of the present drug delivery system, exosomes are considered to be less toxic, safe, and efficient. Recently, exosomes have found great significance as drug carriers and are nano sized (30–150 nm) [103]. Exosomes holding microRNA released from cancer cells have shown to be effectively active targeting agents and biocompatible, which further, may find application in diagnosis and treatment [104]. In recent studies, exosomes modified by peptide functionalization for targeting and drug accommodation capabilities have been used as a drug delivery agent to treat triple-negative breast cancer (TNBC).

The peptide used in the study is mesenchymal–epithelial transition factor (c-met) peptide, which is known to target the overexpressed c-met receptors of TNBC cells [105]. The c-met factor is one of the tyrosine kinase inhibitor receptors responsible for neovascularization and cell endurance. Researchers in the study designed and synthesized macrophage-extracted exosome-conjugated poly (lactic-co-glycolic acid) nanomaterial-loaded DOX (MEP-D)-targeted drug delivery system to treat TNBC. Exosomes derived from macrophages are believed to contain some protein molecules, which would help in cell adhesion and targeting the overexpressed factors in cancer cells. For a cell adhesion and targeting scenario, a c-met factor was found to be overexpressed in TNBC cells, which was able to specifically target the site of TNBC. Furthermore, in vitro and in vivo studies of MEP-D use MDA-MB-231 human breast cancer cell lines in a mouse model. MEP-D showed potential therapeutic agents in vitro and in vivo, with better biocompatibility and efficiency in the treatment of TNBC cells and tumors and awaits further clinical evaluations.

**Figure 5:**
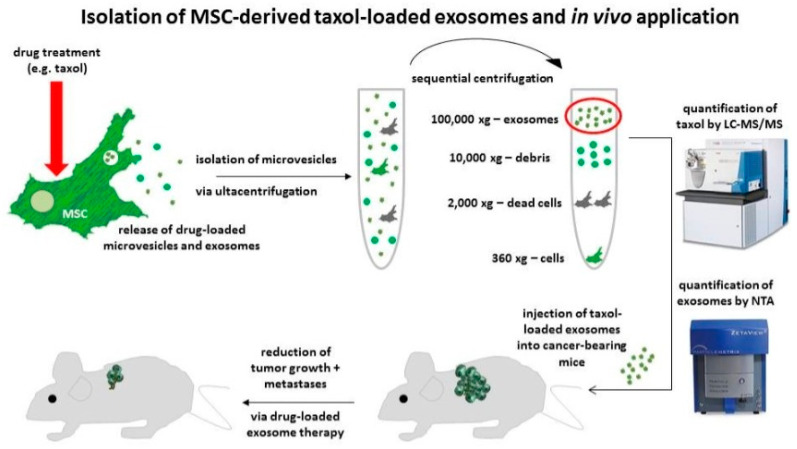
Schematic illustration of isolation of MSC-derived drug-loaded exosomes and efficacy evaluation of targeting agent, in vitro as well as in vivo, to eradicate metastatic breast cancer. Reproduced with permission from ref. [110] Copyright © 2019, MDPI.

Melzer et al. recently extracted exosomes from mesenchymal stem cells (MSCs), which show higher affinity towards CD73, CD90, and CD105 overexpressed factors on the human breast cancer cells. To target these overexpressed factors on cancer cells and tumors, the Taxol drug loaded in exosome-extracted MSCs was utilized as a drug carrier agent and for treatment of tumors in a mouse model. In Figure 5, a schematic representation shows the process of isolation of exosomes from MSCs, which can target specific overexpressed factors, as mentioned above. The loaded drug Taxol and exosomes were quantified before being injected intravenously into mice. Taxol-loaded exosomes were found to be effective and biocompatible against the human metastatic MDAhyb1 cancer cells and tumors. The in vitro and in vivo application of MSC-extracted exosomes for targeting and drug-loading capability and loaded with Taxol (or any suitable drug) would be an effective solution to treat metastatic breast cancer soon and this is awaited for further clinical applications [106,107,108,109,110]. Recent examples of exosome-based drug delivery systems for breast cancer therapy have been summarized in Table 2.

### 3.3. Micelles

The polymeric drug delivery system and treatment in the past four decades have gained significance, especially the drug conjugation with polymers forming covalent bonds between biodegradable polymers and important drugs in use (polymer micelles) to diagnose and treat cancers. The functionalized polymeric micelle design, synthesis, and application in targeting, drug delivery, and treatment of cancers have also gained attention in recent times [114]. The micelle-loaded DOX has been applied to target overexpressed cyclooxygenase-2 (COX-2) factors by using CD44 receptors on breast tumor cells. The micelle-loaded drugs combined with anti-inflammatory drugs may not only reduce inflammation but are also effective in the eradication of metastatic breast cancer by targeting overexpressed COX-2 factors via CD44 receptors in cancerous cells. Otherwise, cubosomes, which have a high membrane surface area and hydrophobic as well as hydrophilic molecular carrier ability nanoparticles, are considered as great drug delivery vehicles [115,116]. The surface modification of cubosomes can increase carried drug accumulation in targeting cancer and decreasing the off-target toxicity. Hyaluronic acid (HA)-modified cubosomes, loading copper acetylacetonate, show potential for treatment of CD44-expression tumors [117].

**Figure 6:**
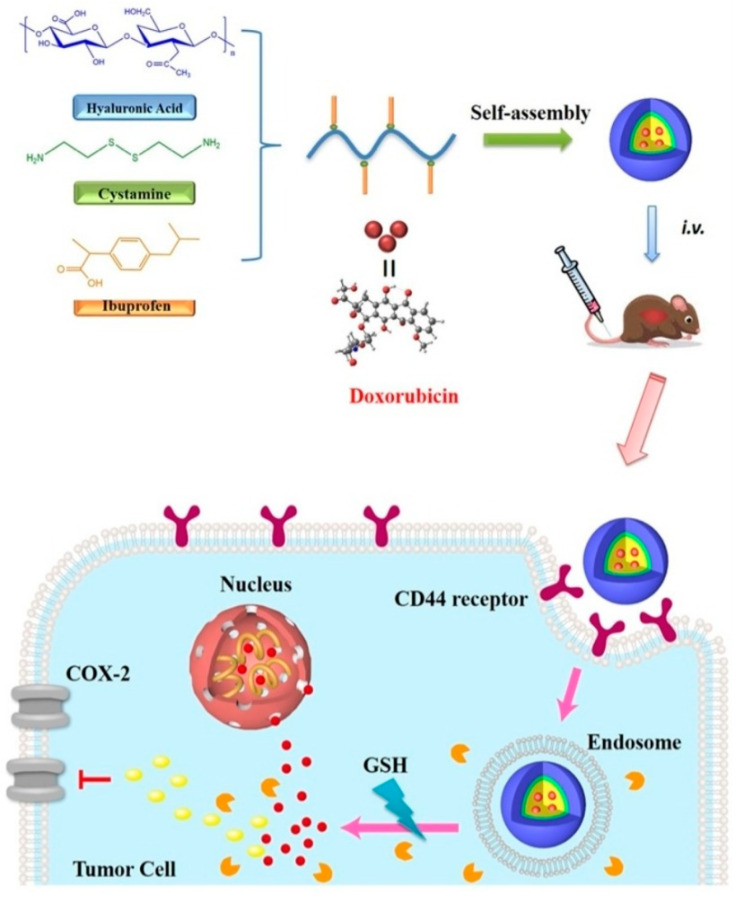
Schematic illustration of a polymeric micelle-based drug delivery system, HA known to target CD44 receptors through COX-2 overexpressed factor on cancer cells and DOX loaded to inhibit breast cancer cells. Reproduced with permission from ref. [118] Copyright © 2020, Elsevier.

In Figure 6, the polymeric micelle was designed and synthesized using cystamine (ss)-functionalized hyaluronic acid (HA-ss) [118]. HA-ss was further conjugated with ibuprofen (BF) and BF, a nonsteroidal anti-inflammatory drug (NSAID), was found to reduce the inflammation caused by the tumor and found effectively biocompatible when conjugated. The polymeric micelle-conjugated BF (HA-ss-BF) underwent self-assembly and was further loaded with DOX to target and treat breast cancer. HA-ss-BF-loaded DOX was found to operate effectively by reduction and oxidation for the controlled released drug in the microenvironment, demonstrating effective results to tumors and showing enhanced biocompatibility in in vitro and in vivo studies. HA-ss-BF-loaded DOX was further evaluated to show high biocompatibility, better drug-loading capacity, and enhanced targeting abilities to target CD44 receptors on breast tumor cells. Furthermore, the delivery efficiency and biocompatibility of this drug delivery system were observed using human cancer cell lines and mouse models in in vitro and in vivo studies, awaiting further clinical evaluations.

**Figure 7:**
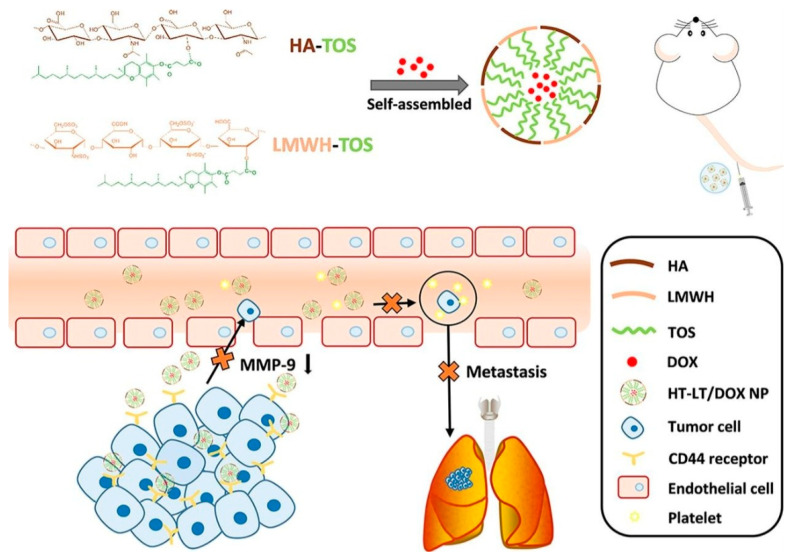
Schematic representation of a polymeric micelle-based drug delivery system, HA known to target CD44 receptors on TNBC cells and drug DOX loaded to inhibit metastasis of breast cancer by downregulating MMP-9 factor. Reproduced with permission from ref. [119] Copyright © 2020, Elsevier.

In recent advances, a study reported an effective drug delivery system befitted with enhanced targeting ability towards overexpressed CD44 receptors TNBC cells and also inhibiting metastases, causing matrix metalloproteinases (MMP) factor [119]. In Figure 7, a peculiar approach to design and synthesis was reported; in the study, hyaluronic acid D-α-tocopheryl succinate (HT) and low-molecular-weight heparin-TOS (LMWH-TOS, LT) were combined to form polymeric micelle HT-LT. Furthermore, HA affinity towards CD44 receptors was exploited to efficiently target the breast tumor site. HT-LT combined further undergo self-assembly to form spherical polymeric micelles loaded with DOX. The DOX-loaded HT-LT was further investigated for the in vitro and in vivo activity and biocompatibility studies. HT-LT-loaded DOX was found to be effective and less toxic towards breast cancer cell lines and 4T1 mouse model, in in vitro and in vivo studies, respectively. For metastatic-breast-cancer-infested mice, the drug carrier of HT-LT loaded with DOX showed effective inhibition of cancer and further inhibited metastases by MMP factors. However, clinical evaluations and verifications are awaited. Recent examples of micelle-based drug delivery systems for breast cancer therapy have been gathered in Table 3.

## 4. Safety and Efficacy Aspects of Lipid-Based Nanomaterials

Since their first development in 1965, liposomes have been successfully developed to become the first nanomedicine-based drug delivery systems to be applied into clinical applications [127]. Liposomes also successfully offer several solutions in the field of cancer immunotherapy, such as: (a) combination therapy: liposomes as an ideal transport for continuing drugs delivery combined with other therapies, such as: phototherapy, chemotherapy, radiotherapy; (b) vaccination: liposomes can improve the delivery system for antigens and other stimulating molecules to T cells or antigen-presenting cells; (c) rewiring the signals of tumors: experts use liposomes to deliver certain drugs to specific cell types to correct or modulate pathways to facilitate better and safer antitumor immune responses; (d) tumor normalization: liposomes can selectively deliver drugs to the tumor microenvironment to overcome immune-suppressive conditions [128]. There are other advantages and benefits of liposomes. After the surface was modified, liposomes were successfully developed using several molecules, such as sialic acid or glycolipids. Liposomes have also been successfully developed into drug-delivery systems in: anticancer, antifungal, anti-inflammatory, and other theranostic fields.

Liposomal doxorubicin has been reported to induce side effects, including hand–foot syndrome (HFS) and acute infusion reaction [129]. To improve the safety quality of liposomal drugs, liposomal drugs are made to be very selective, targeted, and personalized for cancer cells and tissues. In addition, the damage caused by liposomal drugs to normal cells and tissues needs to be minimized. The principle of safety that can be applied in liposomal drugs is a reduction in the use of cytotoxic agents in the clinical setting [130]. Researchers, together with academics, entrepreneurs, and the government also need to develop safety and efficacy-based liposome technology and regulations, considering that liposome-based clinical applications will later be used for humanity and human welfare [131]. The following will explain the latest technology regarding the development of liposomes.

### 4.1. Stealth Liposome Technology

Conventional liposomes have several limitations in multiple aspects, for example: low bioavailability, low binding site, low retention, low efficacy, low specificity, blood clearance, phagocytosis, and opsonization. As a solution to overcome these limitations, scientists developed Stealth Liposome Technology [132]. Stealth Liposome Technology is used as a development drug delivery system. Strand polymers are attached to drug molecules or to a system that can increase the safety and efficacy of various therapeutic agents. There are some significant changes here, especially from the PEGylation process. PEGylation is the most frequent and commonly used modification strategy for formulating long circulating liposomes. PEGylated liposomal drugs show prolonged circulation time and the tumor inhibitory effect is better than normal liposomal drugs and conventional drugs, based on research both in vitro and in vivo [133]. The linkage of covalent liposomes to polyethylene glycol (PEG) protects the active moiety in the recipient immune system, so that antigenicity and immunogenicity are reduced. The changes in physicochemical properties of the active moiety, changes in hydrodynamic size, reduced renal clearance, prolonged circulatory time, hydrophobic and hydrophilic characteristics of the drug, and reduced drug dose frequency were shown but did not reduce efficacy and still showed a decrease in toxicity [134].

### 4.2. Non-PEGylated-Based Liposome Technology

Non-PEGylated liposomes are a drug delivery system that has the potential to be a cancer therapy. This technology makes use of PEGylated liposomes and eliminates the side effects of PEG. Doxorubicin NPL injection provides a better safety profile than conventional therapy [135]. Technology-based liposome agents and products have the potential to increase safety and efficacy, especially anticancer drugs. Nevertheless, extensive and sustainable research is still needed.

### 4.3. Liposomal-Based Products

There are several liposomal-based products under clinical trials (Table 4). If necessary, the impact and effect of the pharmacokinetics, pharmacodynamics, safety profile, and efficacy of liposomal-based products need to be evaluated and investigated regularly. If there are components of liposomes that are thought to affect safety and efficacy, experts and scientists will immediately develop systematic and analytical techniques that are able to measure the concentration of the liposome component [136].

## 5. Conclusions

As in other fields, lipid-based drug delivery systems have significance in cancer-targeted treatment. This delivery system can be considered as a potential treatment therapy in the breast cancer field in the coming era. To increase the efficiency and to be much broader in the application, the retention of the drug and its ability to be stable in the circulatory system is to be improved. The highly efficient conjugation system for the drug to the lipid-based nanomaterials should be studied more thoroughly in order to enter clinical trials and be more precise. To overcome these problems, lipid-based nanomaterials can be combined with polymers, such as PEG, which helps with stability in circulatory fluids, regulated drug release in the estimated time, and helps in targeting the cancer tissue. The polymer combination also improves the half-life and viscosity of the drug and lipid-based nanomaterials. This will lead to efficacy and bioactivity in the drug. Lipid-based nanomaterials, with effective safety from drug toxicity, efficient targeted drug delivery, and drug-loading capacity, will soon be a complete packaged targeted drug delivery system in the effective theranostics of breast cancer.

## Figures and Tables

**Table 1:** Recent examples of liposome-based drug delivery systems for breast cancer therapy.

Vesicular System	Drug/s/API/s	Pathway/Receptor/Targeting Moiety/ Overexpressed Factors/Mode of Action	Progress	Reference
Ligand modified liposome	PTX	SMVTs receptors/biotin and glucose targeting complex	in vitro and in vivo	[91]
Peptide Based liposome	PTX	Folate receptors/Glutamic hexapeptide-folic acid-targeting complex	in vitro and in vivo	[92]
Aptamer Based liposome	DOX	Forkhead box M1 (FOXM1) aptamer	in vitro and in vivo	[93]
Peptide Based liposome	PTX	Glutamic oligopeptide- RGD Peptide targeting moiety	in vitro and in vivo	[94]
Hydrophobic peptide-based liposome	DOX	Hydrophobic penetrating peptide PFVYL1 targeting moiety	in vitro and in vivo	[95]
HA modified Cationic liposome	Honokiol (HNK)	CD44 receptors/HA-liposome-HNK targeting and therapeutic complex	in vitro and in vivo	[96]
Drug-in-cyclodextrin in-liposome (DCL)	17β-Estradiol	Membrane Isolated Steroid Signaling (MISS) pathway/Estrogen α receptors	in vitro and in vivo	[97]
Aptamer based liposome	DOX	AS1411 targeting nucleotide/P-glycoprotein (P-gp) overexpression	in vitro and in vivo	[98]
Drug based liposome	Tamoxifen and Raloxifene	Estrogen and progesterone receptors	in vitro and in vivo	[99]
Drug based liposome	DOX	Protoporphyrin IX nucleus targeting complex	in vitro and in vivo	[100]
API based liposome	N-alkylIsatin	SerpinB2 inhibitor/uPA/uPAR receptors and targeting ligand	in vitro and in vivo	[101]
Drug and API based liposome	PTX, Cetuximab, and Piperine	EGFR inhibition pathways/EGFR receptors	in vitro	[102]

**Table 2:** Recent examples of exosome-based drug delivery systems for breast cancer therapy.

Vesicular System	Drug/s/API/s/RNA/DNA	Pathway/Receptor/Targeting Moiety/ Overexpressed Factors/Mode of Action	Progress	Reference
Drug based exosome	Erastin	Folate overexpressed receptors	in vitro	[111]
Tumor cells derived exosomes	PTX/ Linoleic acid/Cucurbutacin B	CD44 and CD47 receptors	in vitro and in vivo	[112]
MSCs derived exosomes	MicroRNA	LNA-antimiR-142-3p targeting microRNA	in vitro	[113]

**Table 3:** Recent examples of micelle-based drug delivery systems for breast cancer therapy.

Vesicular System	Drug/s/API/s	Pathway/Receptor/ Targeting Moiety/ Overexpressed Factors/ Mode of Action	Progress	Reference
Drug loaded micelle	PTX	F3 targeting peptide/nucleolin overexpression	in vitro and in vivo	[120]
Lipoprotein based micelle	Tetra-O-methyl nordihydroguaiaretic acid (M4N)	LDL receptor, Apolipoprotein B targeting moiety	in vitro and in vivo	[121]
Drug loaded micelle	DTX, Coumarin, Taxotere^®^	Heparin targeting complex and pH based drug delivery system	in vitro and in vivo	[122]
Polymeric prodrug micelle	DOX -P 123 (prodrugs group)	Phenylboric acid-modified F127 tumor-targeting copolymer	in vitro and in vivo	[123]
Drug loaded micelle	Zileuton™	ALOX5 pathway inhibitor of cancer stem cells	in vitro and in vivo	[124]
Stimuli-responsive nano polymeric micelle	PTX	Stimuli-responsive nano polymeric micelle targeting complex	in vitro and ex vivo	[125]
Drug based polymeric micelle	DOX	CD44 and CD24 receptors/ polymeric targeting complex	in vitro and in vivo	[126]

**Table 4:** Liposomal formulation categorized on clinical trial phase and applications.

Products	Stage	References
Liposome-stabilized prostate cancer vaccine	Phase I trials	[137]
Liposome-lipid A-prostate-specific antigen formulation	Phase II trials	[137]
Liposomal anthracyclines (pegylated liposomal doxorubicin, nonpegylated liposomal doxorubicin, and liposomal daunorubicin)	Phase I and phase II clinical trials	[138]
CAF01-adjuvant liposomes as vaccine formulation	Phase I trial	[139]
Vascular targeting cationic liposomes encapsulating paclitaxel (EndoTAG-1 [ET]) for human HNSCC (head and neck squamous cell carcinoma).	Phase I/II clinical trial	[140]
The formulation of liposomal peptides vaccines or plasmid–DNA vaccines	in vivo antigen loading	[141]

## Data Availability

No new data were created or analyzed in this study. Data sharing is not applicable to this article.
